# Arginine and its metabolites stimulate proliferation, differentiation, and physiological function of porcine trophoblast cells through β-catenin and mTOR pathways

**DOI:** 10.1186/s12917-024-04023-w

**Published:** 2024-04-30

**Authors:** Shuai Li, Xiangyang Ye, Xiaolu Wen, Xuefen Yang, Li Wang, Kaiguo Gao, Hao Xiao, Zongyong Jiang

**Affiliations:** Institute of Animal Science, State Key Laboratory of Swine and Poultry Breeding Industry, Key Laboratory of Animal Nutrition and Feed Science in South China, Ministry of Agriculture and Rural Affairs, Guangdong Key Laboratory of Animal Breeding and Nutrition, Guangdong Academy of Agricultural Sciences, Guangzhou, 510640 China

**Keywords:** Arginine, Porcine trophoblast cells, Cell proliferation

## Abstract

Arginine, which is metabolized into ornithine, proline, and nitric oxide, plays an important role in embryonic development. The present study was conducted to investigate the molecular mechanism of arginine in proliferation, differentiation, and physiological function of porcine trophoblast cells (pTr2) through metabolic pathways. The results showed that arginine significantly increased cell viability (*P* < 0.05). The addition of arginine had a quadratic tendency to increase the content of progesterone (*P* = 0.06) and protein synthesis rate (*P* = 0.03), in which the maximum protein synthesis rate was observed at 0.4 mM arginine. Arginine quadratically increased (*P* < 0.05) the intracellular contents of spermine, spermidine and putrescine, as well as linearly increased (*P* < 0.05) the intracellular content of NO in a dose-dependent manner. Arginine showed a quadratic tendency to increase the content of putrescine (*P* = 0.07) and a linear tendency to increase NO content (*P* = 0.09) in cell supernatant. Moreover, increasing arginine activated (*P* < 0.05) the mRNA expressions for *ARG*, *ODC*, *iNOS* and *PCNA*. Furthermore, inhibitors of arginine metabolism (L-NMMA and DFMO) both inhibited cell proliferation, while addition of its metabolites (NO and putrescine) promoted the cell proliferation and cell cycle, the mRNA expressions of *PCNA*, *EGF* and *IGF-1*, and increased (*P* < 0.05) cellular protein synthesis rate, as well as estradiol and hCG secretion (*P* < 0.05). In conclusion, our results suggested that arginine could promote cell proliferation and physiological function by regulating the metabolic pathway. Further studies showed that arginine and its metabolites modulate cell function mainly through β-catenin and mTOR pathways.

## Introduction

Placental growth and development is a process of multiplication, differentiation, invasion, and fusion of porcine trophoblast (pTr2), and the placental plays major physiological roles in immune barrier, substance transport, and factor expression, which are closely related to the characteristics of trophoblast [1–3]. Arginine increased NO and polyamine levels by regulating the mammalian target of rapamycin (mTOR) signaling pathway, and subsequently enhanced cell proliferation [4]. Furthermore, arginine could reduce trophoblast apoptosis, improve placental function and promote fetal development [5]. Arginine also improved the proliferation, differentiation and immune function of porcine trophoblast cells through modulating the activation of mTOR signaling pathway in porcine trophoblast cells [6], which was consistent with Kim’s study [7]. As a source of nitrogen, arginine synthesized a variety of amino acids in vivo, such as ornithine, proline, glutamine and other bioactive molecules, such as NO, polyamine and creatine [8]. Studies had shown that arginine could affect trophoblast cell proliferation, differentiation and apoptosis, which may be related to its metabolites (NO and polyamines). NO and polyamines were reported to stimulate cell proliferation and migration, cellular reorganization, angiogenesis, dilation, increase blood flow, and play an important role in regulating of embryonic development [8]. NO could regulate early embryonic development in pigs [9]. Exogenous addition of the spermidine metabolite putrescine could increase protein synthesis and promote proliferation by activating the mTOR signaling pathway in pTr2 cells [10]. NO can also stimulate follicle-derived gonadotropin secretion [11]. IGF-1 is a major mammalian growth-stimulating hormone that promotes body growth through induction of cell proliferation and regulation of energy metabolism [12, 13]. Arginine has been shown to promote the synthesis and secretion of IGF-1 in vitro and to induce the corresponding signaling cascade [14]. However, the effect of arginine metabolism on pTr2 was still unclear. Therefore, the aim of this study was to investigate the effects of arginine and its metabolites on cell proliferation, differentiation and physiological functions, as well as the effects of mTOR and β-catenin signaling pathways using pTr2 cell model.

## Materials and methods

### Cell culture and treatment

The cell culture was referred to our previous study [15]. Dulbecco’s modified Eagles F12 Ham medium (DMEM-F12) (11,320,033, Thermo Fisher Scientific, MA, USA), fetal bovine serum (FBS) (A3161001C, Thermo Fisher Scientific, MA, USA), and antibiotics were procured from Invitrogen (15,140,122, Thermo Fisher Scientific, MA, USA). Plastic culture plates were manufactured by Corning Inc. (Corning, NY, USA). Unless indicated, all other chemicals were purchased from Sigma-Aldrich (St. Louis, MO, USA). pTr2 cells were seeded and cultured with DMEM-F12 medium containing 10% FBS, 10 mg/mL insulin and 80 U/mL penicillin, and 80 µg/mL streptomycin at 37 ℃ in a 5% CO_2_ incubator. The cells were starved for 6 h, with arginine-free medium. (1) The cells were growth on a basal medium supplied with 0 mM, 0.2 mM, 0.4 mM, 0.8 mM, 1.6 mM, 3.2 mM and 6.4 mM arginine, respectively. (2) The cells were cultured in the basal medium with 0.4 mM arginine (control medium), control medium + 3 mM L-NMMA group, control medium + 3 mM L-NMMA + 0.5 mM SNP, control medium + 5 mM DFMO, control medium + 5 mM DFMO and 10 µM putrescine for 4 days. The cells were treated and collected for the analysis of cell viability, protein synthesis, cell cycle, mRNA, hormone content and free amino acids. L-NMMA (M7033, Sigma-Aldrich, St. Louis, MO, USA), SNP (228,710, Sigma-Aldrich, St. Louis, MO, USA), DFMO (D193, Sigma-Aldrich, St. Louis, MO, USA), putrescine (51,799, Sigma-Aldrich, St. Louis, MO, USA). The concentrations of L-NMMA, SNP, DFMO and putrescine were prepared in accordance with previously studies [16–19].

### Cell viability assay

About 1 × 10^4^ cells per well of pTr2 cells were seeded in 96-well plates and grown as usual. After incubation in 0, 0.2, 0.4, 0.8, 1.6, 3.2 or 6.4 mM arginine medium for 24 h, then 100 µM H_2_O_2_ were added for 4 h. The wells were washed, and fresh basal medium was replaced. Cell Counting Kit-8 (CCK-8) (C0037, Beyotime, Shanghai, China) was added to each well, incubated for 2 h, and read on the spectrophotometer at 450 nm, the measured absorbance is proportional to the number of viable cells [15].

### Flow cytometry analysis

The cellular DNA content and cell cycle was analyzed by flow cytometry. Briefly, about 1 × 10^6^ pTr2 cells were pelleted at 16 000 x g for 5 min. The supernatant was removed and 1 ml of 70% cold ethanol was slowly added during vigorous mixing. Samples were stored at 4℃. Samples were washed twice with phosphate buffered saline (PBS) (BL302A, Biosharp, Beijing, China) and resuspended in PBS containing 150 µg/ml RNase A [15]. DNA was stained with 50 µg/ml propidium iodide for 1 h at 37℃. DNA content was then analyzed by FACS analysis on a Becton Dickinson FACSCanto.

### qPCR analysis

pTr2 cells (1 × 10^5^ cells per well) were seeded in a 6-well plate. After the test, the cells were collected by Trizol (15596026CN, Thermo Fisher Scientific, MA, USA). The protocol of total RNA extraction, quantification, cDNA synthesis and real-time PCR was adapted from the method of Li et al [20]. Briefly, total RNA was isolated from cell samples by using the Trizol method. The prepared 5 µL total RNA was detected by agarose gel electrophoresis with a gel concentration of 1% and 120 V electrophoresis for 30 min. The electrophoresis results were observed in the gel system. RNA concentration and absorbance ratio at 260 nm and 280 nm were measured using a Nanodrop ND-100 nucleic acid protein detector in 1 µL RNA sample. Cells were harvested and homogenized in Trizol solution (15596026CN, Thermo Fisher Scientific, MA, USA). The RNA was reverse-transcribed into complementary DNA in the light of the reverse transcription kit (K1621, Fermentas, Maryland, NY, USA). Gene expression was verified by fluorescence quantitative PCR Kit (RR014A, Takara, Beijing, China). A real-time fluorescence quantitative PCR instrument (Thermo Fisher Scientific, MA, USA) was adopted for detection [21]. Forward and reverse primers (Table 1) were used to amplify the target genes. For quantification, the following conditions of PCR were used: incubation for 10 min at 95 ℃, followed by 40 cycles of denaturation for 15 s at 95 ℃, and annealing and extension for 60 s at 56 to 64 ℃. The mRNA expressions of target genes were calculated by the value of the threshold cycle (Ct) of the real-time PCR as related to that of β-actin using the 2-^ΔΔCt^ method [22], in which ^ΔΔ^Ct = (Ct_gene of interest_ - Ct_β-actin_)treat -(Ct_gene of interest_ - Ct_β-actin_) untreat.

**Table 1:** Primer sequence for qRT-PCR.

Gene Name	Primer Forward (5^,^ -3^,^ )	Primer Reverse (5^,^ -3^,^ )
*Arginase*	CAGAGGAATCGGACAGTGAAGATC	ACCCAGACGAACATTTAGGG
*ADC*	CCTTGGCAGTCAGCATCATC	ATGGTTTCTTCGGCAGGAC
*ODC*	ATCTCTGATGCCCGCTGT	CTGGCTCCGCTATGATTCTC
*iNOS*	ATCTTGGAGCGAGTTGTGGATTGTC	TAGGTGAGGGCTTGGCTGAGTG
*PCNA*	ATCCTGAAGAAGGTGCTGGA	TGAGACGAGTCCATGCTCTG
*EGF*	CATCACATCCTCTTCGCATC	AAGCAGCACTCATCCACGA
*IGF-I*	CCACAGGGTACGGCTCCAG	TGTACTTCCTTCTGAGCCTTGG
*β-actin*	ATCTCACCGACTACCTCAT	TCCTTCCTGATGTCAATGTC

### ELISA

EGF (CSB-E06788p, Cusabio, Wuhan, China), progesterone (CSB-E12869p, Cusabio, Wuhan, China), estradiol (CSB-E06844p, Cusabio, Wuhan, China), hCG (CSB-E05060h, Cusabio, Wuhan, China) and hPL (CSB-E09665h, Cusabio, Wuhan, China) were detected using correspond ELISA kits. Briefly, samples were diluted and added to 96-well microtiter plates coated with antibodies. After incubation for 1 h, wells were washed three times and incubated with biotinylated antibody for 1 h. Plates were washed three times, incubated with streptavidin-HRP conjugate for 30 min, and then chromodeveloping substrate was added. The absorbance at A450-A550 was measured using a microplate reader (Bio-Rad). The concentration was calculated from the standard curve and normalized to the protein concentration of the same sample.

### Urea nitrogen assay

Urea nitrogen was detected by the Urea Nitrogen (BUN) assay kit (Urease method) (C013-2-1, Jiancheng Bioengineering Institute, Nanjing, China), specific determination steps refer to the kit instructions.

### Assay of nitric oxide (NO) content

For NO content determination, total nitric oxide assay kit (S0021S, Beyotime, Shanghai, China) was used. Because NO molecules are unstable, the total NO levels in all test groups were assessed by detecting the content of nitrate and nitrite. All of these measurements were performed according to the manufacturer’s instructions. Data were normalized to the protein concentration [23].

### Detection and quantification of arginine metabolites

The cellular polyamine contents e.g. spermine, spermidine, putrescine was measured using the HPLC method. Briefly, the porcine trophoblast cells were treated with SI-4650 (80 µmol/L) for 48 h, then the cell culture medium was removed. Cells were collected to a new Eppendorf tube and washed with 1.0 mL of PBS (pH 7.4) by centrifugation at 800 rpm at 4℃ for 4 min and discarded the supernatant fluid, then 800 µL cell lysate was added to the tube. After 40 min, the tube was centrifuged at 12,000 rpm for 15 min and the supernatant fluid was transferred into a new 4.0 mL Eppendorf tube. Cell lysate with the same protein content and 20 µL 1,7-diamino-heptane (1 mmol/L) as an internal standard were added into the tube and mixed thoroughly. The mixture was alkalinised by adding 2 mol mL^− 1^ NaOH solution, followed by 10 µL benzoyl chloride. After standing for 20 min under water bath at 40℃, reaction was terminated by adding the saturated sodium chloride solution. Polyamine derivatives were extracted into diethyl ether, followed by evaporating to dryness. The residue was redissolved in 1.0 mL methanol and filtered using 0.22 μm microporous membrane filtration. Protein was determined by BCA assay. HPLC analytical were performed according to the following procedures. Derivative polyamines were separated on a luna C18 column (5 μm, 150 mm×4.6 mm) held at 30℃. The column was eluted with a gradient mixture of acetonitrile (phase A) and water (phase B) at the flow rate of 1 mL min^− 1^. The detection wavelength is 254 nm [24].

### Statistical analysis

Results are expressed as mean ± SEM. All statistical analyses were performed using SPSS software (SPSS Inc., Chicago, IL, USA). The differences among treatments were evaluated using Tukey’s test. Probability values < 0.05 were considered statistically significant.

## Results

### Arginine promoted cell proliferation and physiological functions

**Fig. 1:**
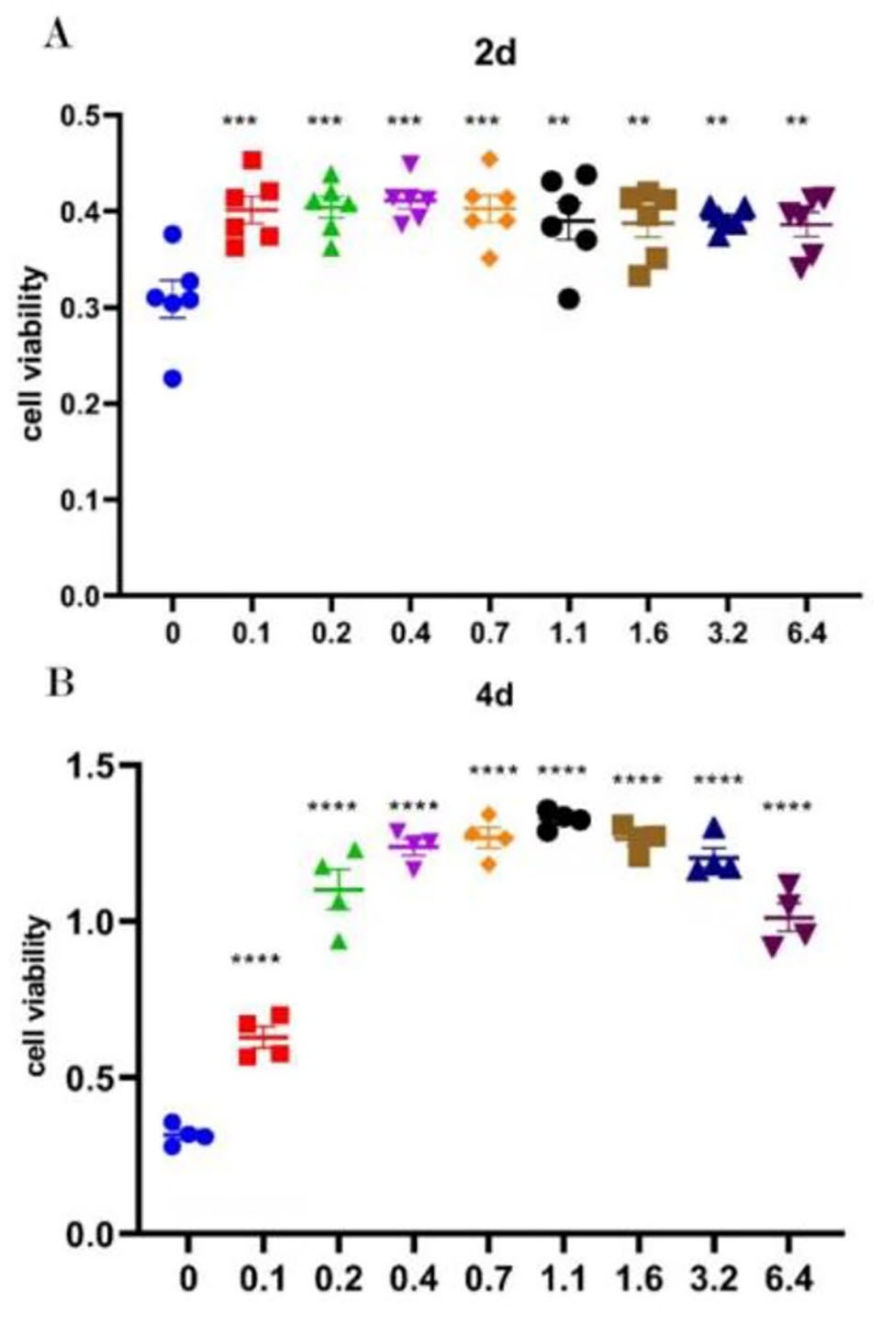
Effect of different concentrations of arginine on the proliferation of porcine trophoblast cells in sows. (**A**) Cell viability on the 2nd day of treatment. (**B**) Cell viability on the 4th day of treatment.

The cell viability was illustrated in Fig. 1. The results showed that arginine significantly increased cell proliferation in a dose-dependent manner on day 2 and day 4 (*P* < 0.05), compared with the control group, and 0.4 mM arginine showed the best promotion effects. To assess the effects of arginine on cell physiological functions, the contents of EGF, progesterone and estradiol were showed in Table 2, while the indexes of cell protein synthesis and urea nitrogen were presented in Table 3. The addition of arginine resulted in a quadratic tendency increase the content of progesterone (*P* = 0.06) (Table 2) and quadratically increased protein synthesis rate (*P* = 0.03), in which the maximum protein synthesis rate was observed at 0.4 mM arginine (Table 3). There was no significant difference in other measured indexes among the groups (*P >* 0.05).

**Table 2:** Effects of different concentrations of arginine on cell physiological functions

	Arginine concentrations (mmol/L)									SEM	*P* value	
	0	0.1	0.2	0.4	0.7	1.1	1.6	3.2	6.4		Linear	Quadratic
EGF (pg/ml)	1.01	1.01	1.45	1.14	1.23	1.28	1.18	1.11	1.23	0.15	0.14	0.24
Progesterone (ng/ml)	0.83	0.94	1.06	1.13	1.04	1.02	1.02	0.98	0.93	0.23	0.17	0.06
Estradiol(pg/ml)	171.49	188.14	190.51	207.13	180.06	196.50	167.64	168.22	167.11	19.78	0.21	0.12

SEM, standard error of the mean (*n* = 4)

**Table 3:** Effects of different concentrations of arginine on cell protein synthesis and urea nitrogen in culture medium

	Arginine concentrations (mmol/L)									SEM	*P*	
	0	0.1	0.2	0.4	0.7	1.1	1.6	3.2	6.4		Linear	Quadratic
Protein synthesis rate (%)	66.46	69.45	72.69	78.43	74.21	70.12	69.18	67.14	65.41	9.71	0.74	0.03
Urea nitrogen(mmol/L)	0.75	0.94	0.66	0.93	0.84	0.62	0.72	0.98	0.93	0.16	0.87	0.76

SEM, standard error of the mean (*n* = 4)

### Arginine promoted the contents of metabolites and gene expressions of metabolic pathway

To investigate the changes in arginine metabolic pathways, we texted the contents of spermine, spermidine, putrescine and NO in the cells and supernatants (Table 4), as well as mRNA expressions of arginine metabolic pathway genes on day 4 (Table 5). Arginine quadratically increased (*P* < 0.05) the intracellular contents of spermine, spermidine, and putrescine as well as linearly increased (*P* < 0.05) the intracellular content of NO in a dose-dependent manner. In cell supernatant, Arginine showed a quadratic tend on putrescine (*P* = 0.07) and a linear tend on NO (*P* = 0.09) (Table 4). Moreover, increasing arginine quadratically increased the mRNA expressions for arginase (*ARG*), ornithine decarboxylase (*ODC*), inducible NO synthase (*iNOS*) and *PCNA* (*P* < 0.05) (Table 5). There was no significant difference in other measured indexes among the groups (*P >* 0.05) (Tables 4 and 5).

**Table 4:** Effects of different concentrations of arginine on the contents of polyamines and NO in cells and supernatants

Item	Arginine concentrations(mmol/L)									SEM	*P* value	
	0	0.1	0.2	0.4	0.7	1.1	1.6	3.2	6.4		Linear	Quadratic
**Intracellular**												
Spermine (µmol/L)	0.54	1.07	1.20	1.24	1.35	1.37	1.20	1.16	0.84	0.13	0.54	0.04
Spermidine (µmol/L)	0.90	1.37	1.45	1.88	1.95	1.87	1.68	1.65	1.05	0.11	0.79	0.03
Putrescine (µmol/L)	0.43	0.80	0.87	1.00	1.64	1.24	1.01	0.93	0.48	0.08	0.63	0.03
NO (µmol/L)	0.52	0.73	0.83	1.35	1.39	1.43	1.52	1.5	1.46	0.29	0.04	0.13
**Cell supernatant**												
Spermine (µmol/L)	0.001	0.001	0.001	0.002	0.002	0.001	0.001	0.002	0.001	0.0001	0.19	0.89
Spermidine (µmol/L)	0.002	0.001	0.002	0.002	0.002	0.001	0.001	0.003	0.002	0.0001	0.14	0.92
Putrescine (µmol/L)	0.706	0.735	0.698	0.957	0.746	1.226	1.604	1.378	1.940	0.081	0.74	0.07
NO (µmol/L)	0.02	0.04	0.08	0.15	0.19	0.26	0.32	0.28	0.23	0.11	0.09	0.17

SEM, standard error of the mean (*n* = 4). NO: nitric oxide

**Table 5:** Effects of different arginine concentrations on mRNA relative expression abundance of enzyme active genes in cells

	Arginine concentrations (mmol/L)									SEM	*P* value	
	0	0.1	0.2	0.4	0.7	1.1	1.6	3.2	6.4		Linear	Quadratic
*ARG*	1.01	1.00	1.43	2.74	1.83	1.45	1.42	1.19	1.03	0.01	0.78	0.05
*ADC*	1.00	1.00	0.86	1.19	0.77	1.11	1.08	1.09	1.02	0.01	0.16	0.69
*ODC*	1.02	1.13	1.44	2.03	1.63	1.40	1.31	1.23	1.09	0.02	0.52	0.04
*i* *NOS*	1.01	1.01	1.31	1.89	1.86	1.65	1.48	1.20	1.12	0.01	0.89	0.001
*PCNA*	1.00	1.00	1.02	1.30	1.27	1.19	1.03	1.09	1.02	0.01	0.25	0.03

SEM, standard error of the mean (*n* = 4). *ARG:* arginase, *ADC*: arginine decarboxylase, *ODC*: ornithine decarboxylase, *iNOS*: induced NO synthase, *PCNA*: Proliferating Cell Nuclear Antigen

### Arginine metabolites promoted cell proliferation and differentiation

**Fig. 2:**
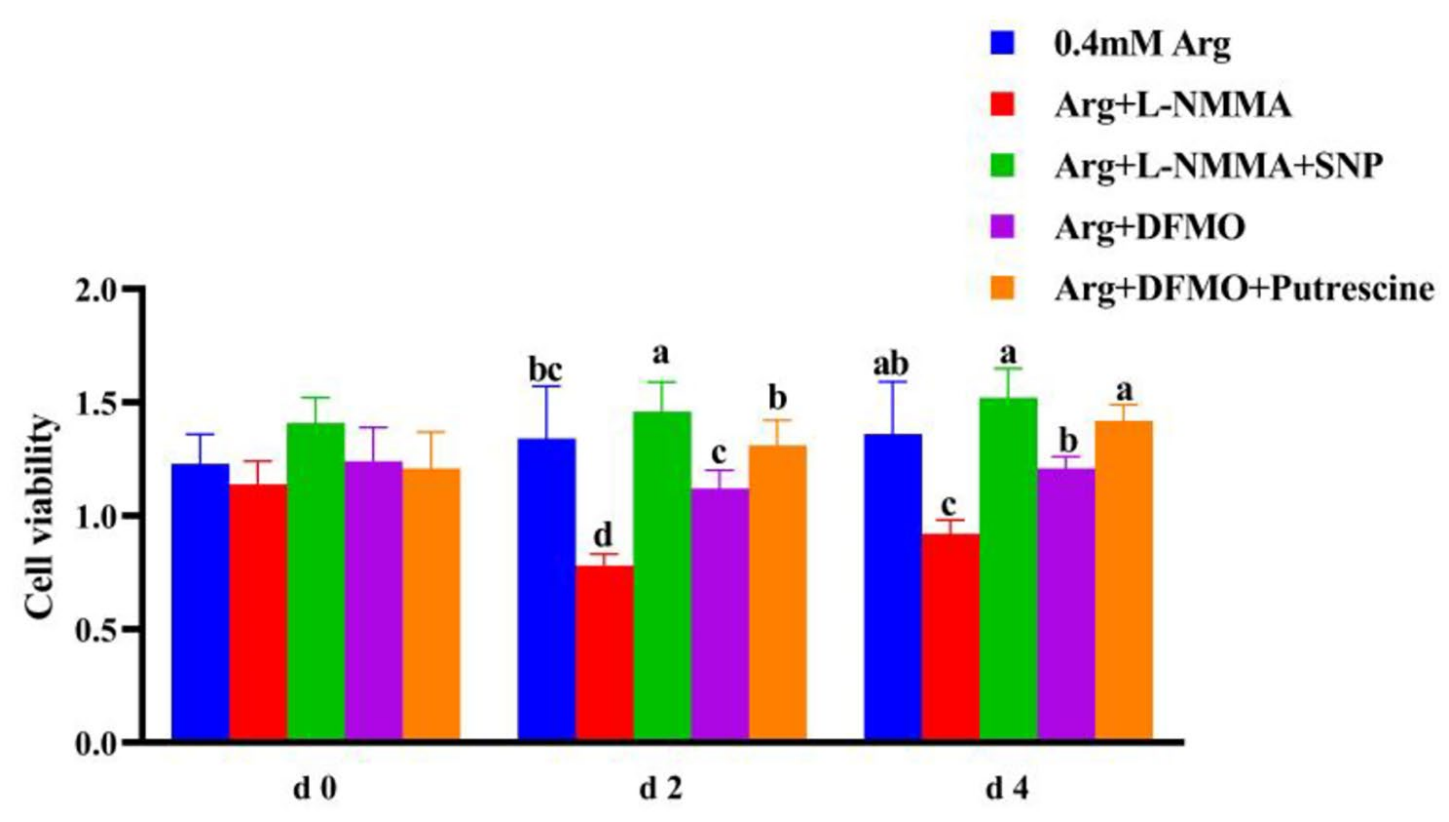
Effects of arginine and its metabolites (NO and polyamines) on the proliferation and differentiation of porcine trophoblast cells in sows. a-c Value columns with different letters are significantly different (*P* < 0.05).

The cell viability was illustrated in the Fig. 2. On day 2 and 4, the addition of L-NMMA inhibited the production of NO synthase and thus inhibited cell proliferation. The cell viability of L-NMMA group was significantly lower than that of control group and SNP addition group (*P* < 0.05), and the addition of DFMO inhibited the production of ornithine decarboxylase, resulting in the failure to generate polyamines, and inhibited cell proliferation, which was restored after exogenously adding putrescine, the cell viability of DFMO group was significantly lower than that of control group and putrescine addition group (*P* < 0.05). In terms of cell cycle, the G1 phase of cells in L-NMMA treatment group was significantly higher than that in other treatment groups (*P* < 0.05), and the proportion of cells in S phase and G2 phase was significantly lower than that in other treatment groups (*P* < 0.05). The addition of SNP provided NO and promoted proliferation, the G1 phase of cells was significantly decreased (*P* < 0.05), while the proportion of cells in S phase and G2 phase was significantly increased (*P* < 0.05). On day 4 of experimental treatment, there were no significant changes in cell cycle and DNA content in all groups (*P* > 0.05) (Tables 6 and 7).

**Table 6:** Effects of arginine and its metabolites (NO and polyamine) on cell cycle

	Cell cycle	0.4mM Arg	0.4mM Arg + 3mM L-NMMA	0.4mM Arg + 3mM L-NMMA + 0.5mM SNP	0.4mM Arg + 5mM DFMO	0.4mM Arg + 5mM DFMO + 10 µM Putrescine
d 2						
	G1	33.37 ± 0.33^bc^	42.16 ± 0.74^a^	23.19 ± 0.91^c^	33.72 ± 0.69^bc^	32.53 ± 0.22^bc^
	S	56.43 ± 0.85^a^	37.89 ± 0.95^c^	46.83 ± 0.68^b^	54.35 ± 0.39^a^	55.40 ± 0.71^a^
	G2	10.20 ± 1.17^c^	19.95 ± 0.66^b^	29.98 ± 1.33^a^	11.93 ± 0.84^c^	12.08 ± 0.70^c^
d 4						
	G1	42.05 ± 0.66	42.96 ± 1.45	36.32 ± 0.73	43.15 ± 1.06	42.02 ± 1.10
	S	37.40 ± 0.77	34.54 ± 0.76	36.24 ± 1.15	37.48 ± 0.50	39.57 ± 0.62
	G2	20.55 ± 1.02	22.50 ± 1.16	27.44 ± 1.09	19.38 ± 0.59	18.41 ± 0.49

SEM, standard error of the mean (*n* = 4). a, b,c means within a row with different superscripts indicate significant

**Table 7:** Effects of arginine and its metabolites (NO and polyamine) on DNA content of cells

	0.4mM Arg	0.4mM Arg + 3mM L-NMMA	0.4mM Arg + 3mM L-NMMA + 0.5mM SNP	0.4mM Arg + 5mM DFMO	0.4mM Arg + 5mM DFMO + 10 µM Putrescine
d 2	21.26 ± 1.09	22.36 ± 0.78	21.70 ± 0.66	22.32 ± 0.62	22.07 ± 0.74
d 4	22.21 ± 0.61	22.33 ± 0.29	22.03 ± 1.09	21.27 ± 0.44	21.08 ± 0.12

**Fig. 3:**
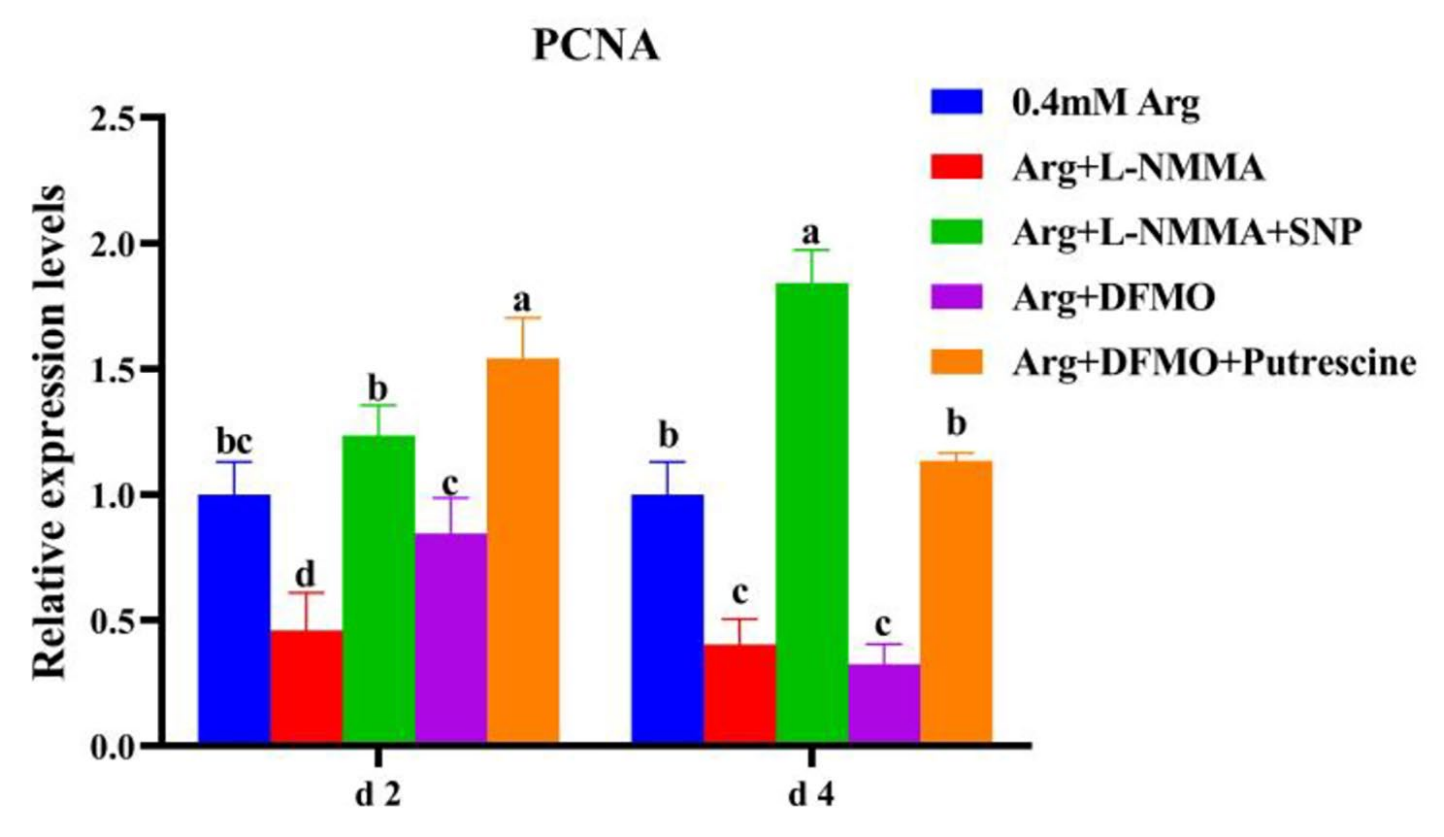
Effects of the addition of sodium nitroprusside and putrescine on mRNA expression of *PCNA* in porcine trophoblast cells. a-c Value columns with different letters are significantly different (*P* < 0.05).

As shown in Fig. 3, on day 2 and 4, the relative expression of *PCNA* mRNA in cells of inhibitor L-NMMA group was significantly lower than control group and SNP treated group (*P* < 0.05). SNP treated group was significantly higher than control group on day 4 (*P* < 0.05), but no significant difference was found on the day 2 (*P* > 0.05). On day 2, the relative expression of *PCNA* mRNA in DFMO group was significantly lower than putrescine supplemental group (*P* < 0.05) but had no significant difference with control group (*P* > 0.05). On day 4, the relative expression level of *PCNA* mRNA in DFMO group was significantly lower than putrescine supplemental group and control group (*P* < 0.05). On day 2 of treatment, the relative expression of mRNA in putrescine treated group was significantly higher than that in control group, but there was no significant difference between putrescine treated group and control group on day 4 (*P* > 0.05) (Fig. 3).

### SNP and putrescine promote secretory factors and physiological functions

**Fig. 4:**
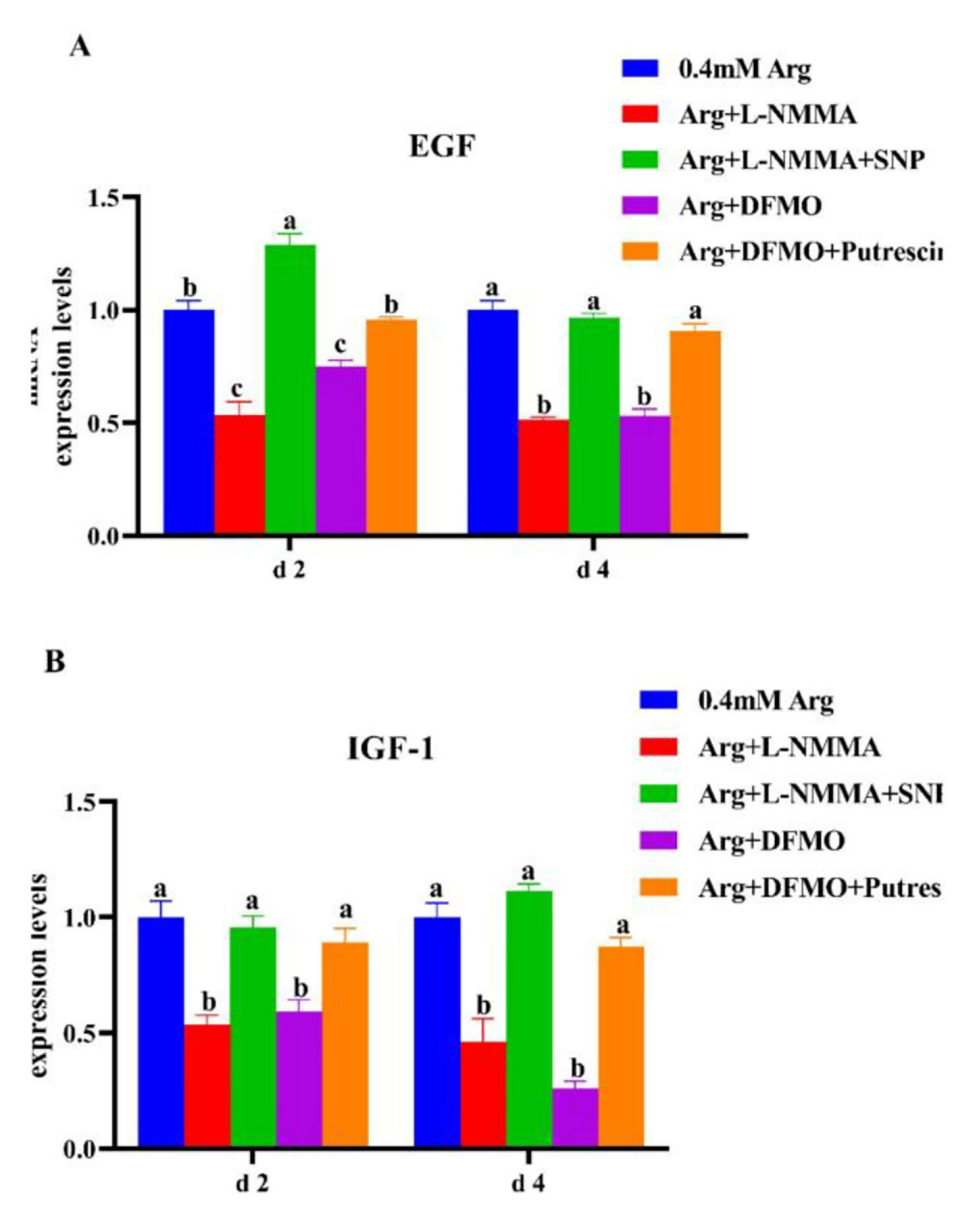
(**A**) Effects of the addition of sodium nitroprusside and putrescine on mRNA expression of *EGF* in porcine trophoblast cells. (**B**) Effects of the addition of sodium nitroprusside and putrescine on mRNA expression of *IGF*-*1* in porcine trophoblast cells. a-c Value columns with different letters are significantly different (*P* < 0.05).

This section may be divided by subheadings. It should provide a concise and precise description of the experimental results, their interpretation, as well as the experimental conclusions that can be drawn. As shown in Fig. 4, on day 2, the relative expression of *EGF* and *IGF*-*1* mRNA in the L-NMMA and DFMO treated cells was significantly lower than that in the control group (*P* < 0.05). the relative expression of *EGF* mRNA in the L-NMMA + SNP group was significantly higher than that in the L-NMMA and control groups (*P* < 0.05), while the relative expression of *IGF*-*1* mRNA was significantly higher than that in the L-NMMA + SNP group (*P* < 0.05), but not significantly different from that in the control group (*P* < 0.05). The relative expressions of *EGF* and *IGF*-*1* mRNA in the DFMO + putrescine group were significantly higher than those in the DFMO group (*P* < 0.05), but not significantly different from those in the control group (*P* > 0.05). On day 4, the relative expression of *EGF* and *IGF*-*1* mRNA in the cells of L-NMMA + SNP group was significantly higher than that of L-NMMA group, but not significantly different from the control group (*P* > 0.05); the relative expression of *EGF* and *IGF*-*1* mRNA in the cells of DFMO + putrescine group was significantly higher than that of DFMO group (*P* < 0.05), the relative expressions of *EGF* and *IGF*-*1* mRNA in pTr2 cells in both L-NMMA and DFMO-treated groups were significantly lower than those in the control group (*P* < 0.05) (Fig. 4A and B).

On both day 2 and day 4, the addition of L-NMMA resulted in significantly lower levels of cellular EGF and IGF-1 secretion than the control and SNP-treated groups (*P* < 0.05), meanwhile, the levels of cellular EGF and IGF-1 secretion in the DFMO-added group were significantly lower than those in the control and putrescine added groups (*P* < 0.05) (Table 8).

**Table 8:** Effects of arginine and its metabolites (NO and polyamine) on cytokine secretion

	Item	0.4mM Arg	0.4mM Arg + 3mM L-NMMA	0.4mM Arg + 3mM L-NMMA + 0.5mM SNP	0.4mM Arg + 5mM DFMO	0.4mM Arg + 5mM DFMO + 10 µM Putrescine
d 2						
	EGF(ng/ml)	2.35 ± 0.20^a^	0.98 ± 0.03^b^	2.64 ± 0.12^a^	1.14 ± 0.21^b^	1.73 ± 0.07^a^
	IGF-1(ng/ml)	191.0 ± 9.93^a^	54.94 ± 3.01^b^	171.43 ± 8.46^a^	87.10 ± 6.62^b^	158.80 ± 9.89^a^
d 4						
	EGF(ng/ml)	2.73 ± 0.08^a^	1.29 ± 0.18^b^	2.89 ± 0.18^a^	1.15 ± 0.11^b^	1.80 ± 0.12^a^
	IGF-1(ng/ml)	198.±11.17^a^	80.38 ± 5.02^b^	149.33 ± 6.27^a^	99.58 ± 4.58^b^	147.19 ± 8.09^a^

SEM, standard error of the mean (*n* = 4). a, b means within a row with different superscripts indicate significant differences (*P* < 0.05)

After day 2, the cellular protein synthesis rate and estradiol content in the group with the addition of L-NMMA were significantly lower than those in the control and SNP-treated groups (*P* < 0.05). The cellular protein synthesis rate, estradiol content, and hCG content in the group with DFMO addition were also significantly lower than those in the control and putrescine-treated groups (*P* < 0.05). There were no significant differences in urea nitrogen and other hormone contents between the groups (*P* > 0.05). On day 4, cellular estradiol content was significantly lower in the L-NMMA-treated group than in the SNP-treated group (*P* < 0.05), while no significant differences (*P* > 0.05) were seen for other hormones such as progesterone and hPL (Tables 9 and 10).

**Table 9:** Effects of arginine and its metabolites (NO and polyamine) on cell protein synthesis and urea nitrogen (d2)

	0.4mM Arg	0.4mM Arg + 3mM L-NMMA	0.4mM Arg + 3mM L-NMMA + 0.5mM SNP	0.4mM Arg + 5mM DFMO	0.4mM Arg + 5mM DFMO + 10 µM Putrescine
Protein synthesis rate (%)	76.10 ± 4.57 ^a^	64.04 ± 3.25 ^b^	72.82 ± 2.14 ^a^	67.71 ± 2.70 ^b^	74.91 ± 3.43 ^a^
Urea nitrogen(mmol/L)	0.93 ± 0.05	0.89 ± 0.02	1.11 ± 0.03	0.81 ± 0.01	0.91 ± 0.02

SEM, standard error of the mean (*n* = 4). a, b means within a row with different superscripts indicate significant differences (*P* < 0.05)

**Table 10:** Effects of arginine and its metabolites (NO and polyamines) on the cytohormone secretion

	Item	0.4mM Arg	0.4mM Arg + 3mM L-NMMA	0.4mM Arg + 3mM L-NMMA + 0.5mM SNP	0.4mM Arg + 5mM DFMO	0.4mM Arg + 5mM DFMO + 10 µM Putrescine
d 2						
	Estradiol (pg/ml)	8.42 ± 0.08^a^	7.78 ± 0.80^b^	9.07 ± 0.40^a^	5.36 ± 0.56^b^	5.18 ± 0.48^b^
	Progesterone (ng/ml)	0.29 ± 0.04	0.29 ± 0.02	0.31 ± 0.03	0.29 ± 0.01	0.28 ± 0.01
	hCG (MIU/ml)	31.87 ± 2.89^b^	32.29 ± 2.08^ab^	36.48 ± 1.09^a^	26.72 ± 1.33^c^	32.01 ± 1.31^ab^
	hPL (ug/ml)	0.20 ± 0.02	0.20 ± 0.01	0.21 ± 0.01	0.20 ± 0.01	0.21 ± 0.01
d 4						
	Estradiol (pg/ml)	5.68 ± 0.1^b^	4.71 ± 0.37^b^	7.14 ± 0.53^a^	5.78 ± 0.59^b^	5.90 ± 0.19^ab^
	Progesterone (ng/ml)	0.32 ± 0.05	0.29 ± 0.01	0.31 ± 0.01	0.29 ± 0.01	0.29 ± 0.01
	hCG (MIU/ml)	34.64 ± 4.37	32 ± 0.74	33.7 ± 1.79	29.37 ± 0.48	32.73 ± 1.01
	hPL (ug/ml)	0.20 ± 0.02	0.19 ± 0.01	0.21 ± 0.01	0.19 ± 0.01	0.19 ± 0.01

SEM, standard error of the mean(*n* = 4). a, b means within a row with different superscripts indicate significant differences (*P* < 0.05)

In summary, the results of this part suggested that blocking cellular NO and polyamine synthesis inhibited cellular hormone secretion, such as estradiol and hCG (*P* < 0.05), and affected cellular physiological functions. Supplementation of NO and polyamine would enhance cellular physiological functions.

## Discussion

In mammals, arginine supplementation promoted embryonic and fetal growth in pigs, rats and sheep [25]. Arginine supplementation in pregnant women reduced the risk of fetal growth disruption due to arginine deficiency in the womb [26]. Arginine positively affected proliferation, migration and signal transduction pathways in sheep trophoblast ectodermal cells. Arginine also provided nutritional support for embryonic growth and development [27]. PCNA was an indicator of cell proliferation and a cofactor for DNA synthase, which was associated with DNA repair and synthesis, PCNA overexpression was often a reliable indicator of tumor progression. It had been reported that arginine can inhibit excessive proliferation of crypt cells in colorectal adenocarcinoma patients, and reduced PCNA expression in these cells [28]. The addition of arginine to dietary kidney-injured rats reduced PCNA expression in glomerular cells [29]. In animal’s body, arginine could be decomposed into a variety of bioactive substances such as NO, ornithine and polyamine, and these metabolic pathways were carried out by ARG, NOS, ADC, ODC and other enzymes. Arginine decarboxylase decarboxylated arginine to agmatine, and ornithine decarboxylase decarboxylated ornithine to putrescine [30]. Arginase regulated the synthesis of arginine and polyamines, converting arginine into urea nitrogen and ornithine, the former protecting cells from ammonia and the latter stimulating cell growth [31]. One of the important factors for a successful sow pregnancy was a constant supply of progesterone, and its concentration directly affected the survival of embryos in the first month of gestation [32]. Studies had shown that the administration of progesterone to pregnant sows could increase the weight and size of the embryos [33]. Both high and low concentrations of arginine increased the plasma progesterone levels in ewes during the late pregnancy [34].

In the current study, we demonstrated that different concentrations of arginine promote the proliferation of porcine trophoblast cells, in the control group, the proliferation of pTr2 cells was almost stagnant due to the absence of arginine. The cell proliferation was promoted with increasing concentrations of added arginine, but the proliferative effect did not increase with the increase of arginine concentration, with 0.4 mM arginine having the most significant promotion effect. The results of this study showed that the addition of different concentrations of arginine significantly increased the relative expression of *PCNA* mRNA in trophoblast cells, thus promoting the proliferation of trophoblast cells in the sow placenta. And the addition of arginine promoted the expression of *ARG*, *ODC*, and *iNOS* mRNA in pTr2 cells, and promoted the arginine metabolic pathway. The content of progesterone in the culture medium showed a quadratic linear increase when different concentrations of arginine were added to the cell medium, indicating that arginine can stimulate the synthesis of progesterone in cells, which had a certain promotion effect on sow pregnancy and embryo survival and growth.

From the above results, it was concluded that arginine could promote the proliferation of pTr2 cells, however, whether this effect was related to its metabolites (NO and polyamines) remains to be confirmed. Therefore, based on the concentration of 0.4 mM arginine, we added the inhibitors of NO and polyamine, and the donor of NO (SNP) and putrescine for further study.

Arginine was converted to polyamines by the action of ornithine decarboxylase and arginase, polyamines played a regulatory role in early mammalian embryogenesis, angiogenesis, trophectoderm and placental growth and development [35]. Polyamines were also scavengers of reactive oxygen species (ROS), protecting DNA, proteins and lipids from oxidative damage [36]. In the porcine placenta, polyamines were synthesized throughout gestation [37]. Arginine could also generate NO under the action of NOS, NO synthesis peaks at day 60 of the sheep placenta, and NOS activity peaks at day 60 of the placenta and remains elevated during gestation [38]. NO stimulated trophoblast cell proliferation, migration and protein synthesis, which also promoted placental angiogenesis by stimulating placental endothelial cells. It was found that the addition of NO in the culture medium of human or sheep placental endothelial cells could activate the mitogen-activated protein kinase pathway to enhance cell proliferation [39]. Studies suggested that maternal secretion of EGF acts synergistically with estrogen to promote rapid placental growth and angiogenesis [40]. IGF-1 also played an important role in cell proliferation and differentiation, organ function, and individual growth and development [41].

The results of this experiment showed that the addition of NOS inhibitor L-NMMA and polyamine synthesis inhibitor DFMO to 0.4 mM arginine, the proliferation of pTr2 cells was inhibited, and the expression level of PCNA mRNA was lower than the normal level. After exogenous addition of SNP (NO donor) and putrescine on basis of the inhibitor, placental trophoblast cell proliferation and PCNA mRNA expression in sows returned to normal. The addition of SNP increases the percentage of cells in G2 and S phases, and promotes division and proliferation. The results indicated that when the synthesis of the metabolites of arginine (NO and polyamines) was inhibited, the proliferation of pTr2 cells was directly reduced, the donor of arginine metabolite NO (SNP) and polyamine substitutes (putrescine) was added exogenously on the basis of the inhibitor, which could restore the proliferation of pTr2 cells to normal, indicating that NO and polyamine had a promoting effect on the proliferation of pTr2 cells. The expression of *EGF* and *IGF-1* mRNA in pTr2 cells was reduced after the addition of the NOS inhibitor L-NMMA and the polyamine synthesis inhibitor DFMO. After treatment with SNP and putrescine on basis of inhibitor, both *EGF* and *IGF*-*1* mRNA expressions were elevated, indirectly indicating that the metabolites NO and polyamines could promote the synthesis of EGF and IGF-1 in pTr2 cells, and it played an important role in the growth and development of the placenta. However, due to the lag of mRNA relative expression, protein expression is the most indicative factor, so we will further study the expression and function of proteins in the future.

## Conclusions

In summary, different concentrations of arginine could promote the proliferation of pTr2 cells, with the best effect at a dose of 0.4 mM. Arginine significantly increased the content of polyamines and NO in cells, and increased the concentration of free ornithine in the cell supernatant. Treatment of pTr2 cells with 0.4 mM arginine significantly increased the expression of *PCNA*, *ODC* and *iNOS*, and promoted the rate of protein synthesis in cells. In this study, by adding inhibitors and donors, we demonstrated that NO and polyamines, the metabolites of arginine, could regulate the cell cycle, increase the expression of *PCNA*, *EGF* and* IGF*-*1*, promote the production of estradiol and hCG in cells. The pathways related to cell proliferation and transporters need to be further investigated.

## Data Availability

All the datasets used and analyzed during the current study are included in the manuscript.

## Abbreviations

ARG: arginase
DFMO: difluoromethylornithine
EGF: epidermal growth factor
FBS: fetal bovine serum
hCG: human chorionic gonadotropin
IGF-1: Insulin-like growth factor-1
iNOS: inducible nitric oxide synthetase synthase
L-NMMA: NG-Monomethyl-L-arginine
mTOR: mammalian target of rapamycin
NO: nitric oxide
NOS: nitric oxide synthetase
ODC: ornithine decarboxylase
ODC: ornithine decarboxylase
PCNA: proliferating cell nuclear antigen
PCR: polymerase chain reaction
pTr2: porcine trophectoderm cells
qRT-PCR: quantitative real-time PCR
ROS: reactive oxygen species
SNP: sodium nitroprusside
